# Supplementary material for: Provider perspectives on the use of motivational interviewing and problem-solving counseling paired with the point-of-care nucleic acid test for HIV care
Source: PLoS One. 2022 Jun 22;17(6):e0270302. doi: 10.1371/journal.pone.0270302 (PMC9216572; doi:10.1371/journal.pone.0270302)
Supplement: Attachment — Submitted filename: Reviewer Comments + Response_2022-02-22.doc [file pone.0270302.s002.doc]

*Owned by King County and Managed by the University of Washington*

February 14, 2022

Re: PONE-D-21-32892

Dear PLOS ONE Editorial Committee,

Thank you for the opportunity to revise and resubmit our manuscript entitled, “Provider Perspectives on the Use of Motivational Interviewing and Problem-Solving Counseling Paired with the Point-of-Care Nucleic Acid Test for HIV Care (GAIN Provider).” We have responded below to the two reviewers’ comments, which we feel have made this a stronger paper. We have noted the reviewer comments in bold and our responses in plain font.

Please let us know if we can provide any further details to facilitate your review of this manuscript for publication in PLOS ONE.

Sincerely,

Dana Lee Atkins, MPH

University of Washington

**Reviewer #1**

**Thank you for the opportunity to review this manuscript. It addresses an important context. I provide recommendations for improvement that could strengthen its relevance and impact**.

**Introduction:
Paragraph 2/3/4. PSC or problem solving therapy- also successfully used to address alcohol use and HIV adherence. Apart from the STRIVE trial, there are two trials on MI-PST among PLWH in South Africa that have just been completed and that show MI-PST is feasible to deliver, acceptable to patients and providers and has promising outcomes. I’ve added references to the trial protocols and formative/process evaluation work here.**

- **Myers, B., Parry, CDH, Morojele, N., Nkosi, S., Shuper, P., Kekwaletswe, C., Sorsdahl, K. (2020). “Moving forward with life’: Acceptability of a brief alcohol reduction intervention for people receiving antiretroviral therapy in South Africa. International Journal of Environmental Research and Public Health. 17(16):5706. doi: 10.3390/ijerph17165706.**
- **Myers, B., Lund, C., Lombard, C., Joska, J., Levitt, N., Butler, C., Cleary, S., Naledi, T., Milligan, P., Stein, D., Sorsdahl, K. (2018). Comparing dedicated and designated models of integrating mental health into chronic disease care: study protocol for a cluster randomized controlled trial. BMC Trials, 19: 185. DOI: 10.1186/s13063-018-2568-9.**
- **Parry, C.D., Morojele, N.K., Myers, B.J., Kekwaletswe, C.T., Manda, S.O.M., Sorsdahl, K., Ramjee, G., Hahn, J.A., Rehm, J., & Shuper, P.A. (2014). Efficacy of an alcohol-focused intervention for improving adherence to antiretroviral therapy (ART) and HIV treatment outcomes - a randomised controlled trial protocol. BMC Infectious Diseases, 14: 500. doi:10.1186/1471-2334-14-500.**
- **Petersen Williams, P., Brooke-Sumner, C., Joska, J., Kruger, J., Vanleeuw, L., Dada, S., Sorsdahl, K., Myers, B. (2020). Young South African Women on Antiretroviral Therapy Perceptions of a Psychological Counselling Program to Reduce Heavy Drinking and Depression. Int. J. Environ. Res. Public Health, 17, 2249. https://doi.org/10.3390/ijerph17072249.**

We appreciate the reviewer pointing to these other bodies of literature. Three of the four most pertinent studies/protocols have been included in the 4^th^ paragraph of the introduction.

**For the third paragraph- perhaps given an example or two of non-motivational factors that impact on adherence- I would suggest highlighting mental health and substance use concerns, especially as the PST literature is very much focused on the benefits of helping people develop adaptive problem solving and coping skills to manage negative emotions and avoid substance use coping.**

A sentence has been included in paragraph 4 of the introduction to include how mental health and substance abuse challenges impact ART adherence. The following sentence has been included:

*“In addition, a combination of both MI and PSC has been shown to be effective and acceptable in reducing substance use in emergency department patients (11) and young women (12) in South Africa, where both mental health and substance abuse share a direct link to ART adherence challenges. Additionally, two randomized trials are ongoing in South Africa to further illustrate the efficacy of MI and PSC in ART adherence for populations struggling with alcohol abuse and depression (11,13).”* **Please can you revise the final paragraph of the introduction so that the rationale for conducting this study, the contribution to the literature and the specific aims of this paper are clear.**

Thank you for this feedback. The last paragraph has been edited to better explain the aims and contribution to the literature and future practical real-world implications this study has. The last paragraph now reads:

*“As part of the formative work for the GAIN Study, this qualitative study aimed to gather medical provider perspectives on the feasibility, acceptability and formative development of a brief POC NAT-based adherence and MI or PSC intervention for Madison Clinic providers to use during clinic visits. Through the findings of this qualitative study, we seek to gather further information for successful implementation of the GAIN study and future dissemination of the POC NAT, MI and PSC into real-world clinical practice in HIV clinics in the United States.”*

**Materials and methods: Overall the methods needs to be fleshed out to comply with standard reporting guidelines for qualitative research**

1. **Please ensure that you follow the COREQ or similar reporting guidelines for qualitative research and include the appropriate checklist as a supplementary file.**

Reference to the COREQ reporting guidelines has been added as the last sentence in the Introduction and the checklist has been included as a Supplement 1.

2. **Under study design and population, in keeping with COREQ guidelines, a description of the design needs to be explicitly made. Here it would be appropriate to note that the formative work is embedded in a broader study. In terms of setting, it would be useful to describe and explain the kinds of clinics offered- ie what is a moderate needs clinic and how does it differ from other levels of need?**

Thank you for this feedback. The paragraph now reads to include study design, that the formative work is embedded in a broader study, and that the moderate needs clinic caters towards people who experience adherence challenges due to socioeconomic, mental and addiction challenges. The paragraph now reads:

*“Between July and September 2020, this qualitative study recruited medical providers from the Madison clinic, a Ryan White-funded clinic at Harborview Medical Center where the larger GAIN Study that this project was embedded in will enroll. This qualitative work followed the COnsolidated criteria for REporting Qualitative research (COREQ) guidelines (Supplement 1). We used content analysis design and convenience purposive sampling in order to obtain a diversity of viewpoints from providers with various durations of providing HIV care and diversity in gender and race/ethnicity. We oversampled physicians providing care in the moderate needs (“Mod”) clinic within the Madison clinic due to this clinic catering towards patients that oftentimes are experiencing adherence challenges attributed to socioeconomic, mental and addiction difficulties. A total of 11 in-depth interviews (IDI) with HIV care providers were conducted, however only 10 were recorded and therefore included in the analysis (Table 1).”*

3. **Who developed the IDI guide and was it piloted?**

The IDI guide was developed by the study team, which includes members who are experts on ART adherence, MI and PSC, and HIV testing, including a care provider who previously practiced at the clinic. The IDI guide was not piloted to additional providers. Check-ins and debriefing discussions were done after interviews to ensure that both interviewers present during the IDI felt that the question guide was eliciting robust answers and that no additional questions were needed.

4. **Please explain the process of recruiting providers, obtaining their consent to participate. How were they approached and by whom? Did anybody refuse to participate and if so why?**

The PI reviewed the list of medical providers at the clinic and, over the course of two weeks, sent an email to approximately 20 providers, with special attention paid to representation of diverse age and experience, genders, and race/ethnicity. This email included information about the study and the option to opt in by contacting the study coordinator. Interviews were scheduled and verbal consent was obtained by the study coordinators, who had limited previous contact with these medical providers. No providers refused to participate at the time of consent, and no information was collected from providers who did not opt in from the original email.

We have added the following text

*“Providers were recruited by the study Principal Investigator (JDS) via email and asked if they would be interested in participating in an IDI. Interested providers were then scheduled by study staff (LV, LN) for a time that worked best for the provider to conduct the interview. IDIs were conducted by study staff (LV, LN), audio recorded, and transcribed verbatim (DLA, LV, LN).* *Verbal consent was collected using an IRB-approved information statement.”*

5. **More information is needed about the qualifications and background of the staff who conducted the interviews as per COREQ guidelines.**

Thank you for this feedback. Both interviewers hold MPHs and have prior experience in qualitative data collection. I have added in their qualifications/background to the paper. LRV holds an MPH and LN holds a MPH and MSW.

6. **I am struggling to see the relevance of the 5 a’s framework for HIV counselling- please can you provide more information on why this and not another framework was chosen. At a minimum this framework needs to be described much more fully, possibly in the background as a a way of trying to understand how MI-PST approaches can be integrated into the various components of counselling.**

Thank you for this feedback. The Five A’s framework is described in the data analysis and collection section of the manuscript. A sentence has been added to further explain why this framework was used. Additionally, further description of the framework is cited in the Discussion section of the paper. The pertinent sentences now read:

*“Transcripts were analyzed using directed content analysis guided by an adapted Five A’s framework.(18) The Five A’s model is based on the trans-theoretical model of behavior (19); it was originally developed by the US Department of Health and Human Services to encourage smoking cessation (20) and has since been adapted to obesity management (21–23). For our purposes, we used the Five A’s framework to analyze providers’ current adherence counseling practices due to its successful use in eliciting behavioral changes in chronic disease management.”*

7. **Was there any member checking of the themes?**

Themes were agreed upon by the three coders (DLA, LV and LN) and then confirmed applicability was verified by group discussion with the lead researchers, DR and JDS.

**Results
Please provide a summary of the main themes that emerged from the IDIs and how these relate to each other.**

The main themes are now listed in the last sentence of the first paragraph of the results section. A sentence was added that reads:

*“The main themes that emerged from the data included: current assessment and advising practices, use of the POC NAT for adherence assessment, incorporation of the POC NAT into clinic flow, assessing patient adherence using MI or PSC, advising patients using MI or PSC, assisting patients with adherence challenges using MI and PSC, and incorporating counseling into clinic practice.”*

**Reading through the results, I think there is an opportunity to present these more critically. For instance, in the first theme, it is clear that psychosocial problems impact on adherence, but in the second theme, the providers are not advising patients on how to manage these challenges, but are focused on “telling” patients to be adherent. So it is noteworthy that they do not currently use a patient-centred *[centered]* approach. A lot of the results can be streamlined and stated more succinctly so the narrative flows through the themes.**

Thank you for this thoughtful feedback. A couple sentences have been added throughout the Results section to better succinctly narrate through the themes. **Discussion. I have few comments here, except for a reflection on** **the importance of supervision and support when training providers in MI-PST techniques. Ongoing peer and group supervision can [h]elp providers develop competency and confidence in their practice beyond a single workshop and increases the likelihood that they will use the skills. It is also a vehicle for brainstorming challanges[challenges]/resources with colleagues.**

**This article reflects on this:
Jacobs, Y., Myers, B., van der Westhuizen, C. et al. Task Sharing or Task Dumping: Counsellors Experiences of Delivering a Psychosocial Intervention for Mental Health Problems in South Africa. Community Ment Health J 57, 1082–1093 (2021).** [**https://doi.org/10.1007/s10597-020-00734-0**](https://doi.org/10.1007/s10597-020-00734-0)

Thank you for the reference. The topic of supervision and continued training has been added to the discussion section with this reference.

**Reviewer #2:**

1. **In the first paragraph, the last line of the of the introduction section, it states: “One strategy for adherence monitoring and support is provider counseling”. What the authors mean by “provider counseling”? It is counseling for providers? Or counseling by providers? In both cases I couldn’t attend well.**

Thank you for pointing out the confusion in the wording of the last sentence. It now reads as “provider-led counseling”.

**Methods

Dear Editor, thank you for inviting this manuscript. It is important in that it is combined with PSC to improve adherence.**

1. **“Following consensus coding, the remaining nine transcripts were independently coded by one member of the coding team. All transcripts were then coded by another member of the team, and disagreements in code application were noted.” In the first sentence, I understand that the coding of the remaining transcripts was done by one of the member[s] of the team. In the second sentence, I could understand that it was done by another member of the team. Would the authors clarify this?**

Coding was done by all three (each person reviewed three of the nine remaining transcripts) and then these were swapped to another person who reviewed someone else’s original three. To ensure clarity in how the coding process was completed, the section of the paragraph that was confusing now reads,

*“Following consensus coding, the remaining nine transcripts were divided equally amongst the three coders and individually coded. Transcripts were then traded and re-coded by another member of the team. Disagreements in code application and were resolved through group discussion with the larger team.”*

2. **I was looking for the description about the MI and the PSC in the methods though I couldn’t. how the authors implemented the MI and the PSC? For how many sessions? At what setting? For how long? This should have been stated so that the readers can judge about the basics of the interventions. If the authors have another paper in this aspect, it should be cited and linked.**

Thank you for your question. MI and PSC have not yet been implemented in the study. This formative qualitative component sought to explore preferences, thoughts and opinions on the use of MI or PSC in the larger GAIN study that had not begun. . We discuss how this qualitative study is a part of the larger GAIN study in the introduction. For clarification purposes, the last pertinent sentences of the last paragraph now read: *“The GAIN Study is a Centers for Disease Control and Prevention (CDC) funded study that will evaluate the implementation of POC NAT combined with either MI or PSC counseling strategies in a clinic setting and a community setting in Seattle, WA. As part of the formative work for the GAIN Study, this qualitative study aimed to gather medical provider perspectives on the feasibility, acceptability and formative development of a brief POC NAT-based adherence combined with either MI or PSC counseling techniques as an intervention for Madison Clinic providers to use during clinic visits.”*

**Results**

3. **In the main results section, it looks that authors reported the findings using thematic topics: ex: Current Assessment and Advising Practices, Creation of the GAIN model, Evaluating the Point-of-Care NAT Intervention Using the Five A’s Framework, Assess: Using the Point-of-Care NAT for Adherence Assessment, Arrange: Incorporating POC NAT into Clinic Flow, etc. However, the results section of the abstract does not contain at least some of these topics.**

Thank you for highlighting this incongruency. The results of the abstract now include wording that mirrors some of the thematic topics. The results in the abstract now reads,

*“****Results:*** *Providers favored the use of a combined adherence counseling technique and POC NAT for their non-adherent patients living with HIV. Providers believed the intervention was an improvement on current assessment and advising practices. However, concerns about extended wait times for the POC NAT results influenced perceptions about feasibility around clinic flow and incorporation into clinic practice. Providers believed that acceptability of POC NAT implementation would be enhanced by including a subset of patient populations whom tend to be in the clinic for longer periods, and in tandem face greater ART adherence challenges”.*

Additionally, minor changes have been made throughout the abstract to comply with the 300 word limit.

4. **What is “SAMABA [SAMBA] II”?**

The SAMBA II stands for “Simple Amplification Based Assay version II (SAMBA II) Semi-Q POC NAT”. The first time the term “SAMBA II” is used, this definition is provided. It is the second sentence under the subsection “Evaluating the Point-of-Care NAT Intervention Using the Five A’s Framework” in the results section.
